# AAPM task group 241 – MR-guided focused ultrasound

**DOI:** 10.1186/2050-5736-3-S1-O70

**Published:** 2015-06-30

**Authors:** Keyvan Farahani, Jason Stafford, Rajiv Chopra

**Affiliations:** 1 National Institutes of Health, Bethesda, Maryland, United States; 2 MD Anderson Cancer Center, Houston, Texas, United States; 3 University of Texas Southwestern Medical Center, Dallas, Texas, United States

## Background/introduction

Task Group (TG) 241 of the American Association of Physicists in Medicine (AAPM), operating under the Work Group for Technology Assessment in Image-Guided Interventions, was formed in September of 2013 with an international membership of experts from MR-guided focused ultrasound (MRgFUS), focused ultrasound and ultrasound physics, radiation oncology, industry and federal agencies.

The charge of TG-241 is to report through peer-reviewed publications on the assessment of state-of-the-art clinical MRgFUS technology, including the intrinsic system characteristics, quantitative metrics, sources of uncertainty, quality assurance measures, data types and nomenclature as well as training issues for medical physicists.

## Methods

In addition, through related sub-group activities, TG-241 will consider development of an open tool as a public resource for the MRgFUS research community.

## Results and conclusions

The goal of this presentation is to introduce TG-241 to the research community and solicit input about its current activities and future directions.

